# Genomic resources in mungbean for future breeding programs

**DOI:** 10.3389/fpls.2015.00626

**Published:** 2015-08-10

**Authors:** Sue K. Kim, Ramakrishnan M. Nair, Jayern Lee, Suk-Ha Lee

**Affiliations:** ^1^Department of Plant Science and Research Institute of Agriculture and Life Sciences, Seoul National UniversitySeoul, South Korea; ^2^AVRDC-The World Vegetable Center South AsiaHyderabad, India; ^3^Plant Genomics and Breeding Institute, Seoul National UniversitySeoul, South Korea

**Keywords:** mungbean, genetic resource, genetic diversity, breeding, molecular and genomics approaches, mungbean improvement

## Abstract

Among the legume family, mungbean (*Vigna radiata*) has become one of the important crops in Asia, showing a steady increase in global production. It provides a good source of protein and contains most notably folate and iron. Beyond the nutritional value of mungbean, certain features make it a well-suited model organism among legume plants because of its small genome size, short life-cycle, self-pollinating, and close genetic relationship to other legumes. In the past, there have been several efforts to develop molecular markers and linkage maps associated with agronomic traits for the genetic improvement of mungbean and, ultimately, breeding for cultivar development to increase the average yields of mungbean. The recent release of a reference genome of the cultivated mungbean (*V. radiata* var. *radiata VC1973A*) and an additional *de novo* sequencing of a wild relative mungbean (*V. radiata* var. *sublobata*) has provided a framework for mungbean genetic and genome research, that can further be used for genome-wide association and functional studies to identify genes related to specific agronomic traits. Moreover, the diverse gene pool of wild mungbean comprises valuable genetic resources of beneficial genes that may be helpful in widening the genetic diversity of cultivated mungbean. This review paper covers the research progress on molecular and genomics approaches and the current status of breeding programs that have developed to move toward the ultimate goal of mungbean improvement.

## Introduction

There is great interest in genetic and genomic analyses of mungbean (*Vigna radiata* (L.) R. Wilczek) due to this crop’s valuable nutritional and health benefits, especially in developing countries, where malnutrition is a major issue. Mungbean represents a cheap source of carbohydrates and high-quality protein, folate, and iron. Mungbean, a warm-season leguminous species, has a short life cycle (approximately 60 days) and is mainly cultivated on small farms in South, East, and Southeast Asia (Kang et al., 2014). As mungbean roots fix atmospheric nitrogen through symbiosis with nitrogen-fixing rhizobia, this crop is valuable both economically and nutritionally because it can improve soil quality and reduce the amount of nitrogen fertilizer required in the soil when grown in rotation with cereals, thereby increasing cereal grain and straw yields due to its residual effects (Yaqub et al., 2010). Mungbean flowers in response to short day conditions; however, it grows in frost-free areas within a wide range of latitudes, as its optimal temperature is greater than 15°C (Imrie, 1996). As a result, mungbean has an extensive cultivation area, from Asia to Africa, South America, and Australia (Anonymous, 2010). However, considering the flexibility of growth conditions, the yield and harvesting index of mungbean are low due to its non-synchronous maturity (Bushby and Lawn, 1992) and the effects of biotic and abiotic stresses (Chotechuen, 1996).

The popularity of mungbean has been increasing throughout the world; mungbean was selected by AVRDC-The World Vegetable Center (AVRDC) as an iron-rich food source to fulfill nutritional needs (Vijayalakshmi et al., 2003). Approximately 3.5 billion people living in developing countries are suffering from iron deficiency, the most common micronutrient disorder, which eventually progresses into anemia (ACC/SCN, 2001). Various approaches have been formulated to combat this problem, among which the dietary approach is the most promising for sustainable, long-term success (Yang and Tsou, 1998; ACC/SCN, 2001). Mungbean can be processed into flour, soups, porridge, noodles, and ice cream, making it highly versatile for the human diet. Mungbean dishes are eaten daily throughout Asia, including the traditional Indian porridge dhal, the Thailand noodle salad Yum Woon Sen, and the Korean sprout side dish Sukjunamul. In addition, mungbean forage is beneficial in the diet of sheep, without any adverse effects (Garg et al., 2004), and the haulms are used as livestock feed (Agboola and Fayemi, 1972). As a consequence, the global consumption of mungbean has increased by 22–66% from 1984 to 2006 (Shanmugasundaram et al., 2009), and its annual production also has increased by a large percentage. With a cultivation area of about 6 million hectares (Nair et al., 2012), the largest mungbean cultivation occurs in Asian countries including India, China, Myanmar, and Indonesia, accounting for ∼90% of world production (Lambrides and Godwin, 2007). Currently, mungbean is considered to be a major cash crop, and mungbean research programs are being conducted globally. For example, the Ministry of Agriculture in Thailand has been working on producing mungbean cultivars that produce higher yields, resist diseases and insects, and provide high-quality seeds (Vayupharp and Laksanalamai, 2013). Efforts are in progress to develop an International Mungbean network to coordinate the research activities between the different research groups (AVRDC, 2015).

Mungbean belongs to the subgenus Ceratotropis in the Leguminosae genus *Vigna*, the tribe *Phaseoleae*, and the family *Fabaceae*. The subgenus Ceratotropis consists of 23 species (Tomooka et al., 2002, 2010; Aitawade et al., 2012), including widely cultivated Asian species such as moth bean (*V. aconitifolia*), adzuki bean (*V. angularis*), black gram (*V. mungo*), créole bean (*V. reflexo-pilosa* var. *glabra*), jungle bean (*V. trilobata*), Tooapee (*V. trinervia*), and rice bean (*V. umbellata*; **Table 1**). Their genome sizes vary from 416 to 1,394 Mb (Parida et al., 1990); *V. radiata, V. mungo*, and *V. angularis* have relatively small sizes of 579, 574, and 538 Mb, respectively (Arumuganathan and Earle, 1991).

**Table 1:** Cultivation areas of Asian *Vigna* germplasms and their genomic resources.

Species name	Common name	Chromosome number	Origin	Cultivation areas	Genome sequence availability	Reference
*Vigna aconitifolia*	Moth bean	2n = 2x = 22	South Asia	India and the Far East	Not available	Adsule (1996)
*Vigna angularis*	Adzuki bean	2n = 2x = 22	East Asia	China, Japan, Korean peninsula	Available (Kang et al., 2015)	Kaga et al. (2008)
*Vigna mungo*	Black gram	2n = 2x = 22	South Asia	South and Southeast Asia	Not available	Gupta et al. (2013)
*Vigna radiata*	Mungbean	2n = 2x = 22	South Asia	South, East and Southeast Asia	Available (Kang et al., 2015)	Nair et al. (2012)
*Vigna reflexo-pilosa*	Creole bean	2n = 4x = 44	Southeast Asia	Vietnam, Philippines (as pulse); India, Mauritius, and Tanzania (as forage)	Not available	Tomooka et al. (2002)
*Vigna trilobata*	Jungle bean	2n = 2x = 22	South Asia	Africa, Australia, Madagascar, Mauritius, and South America	Not available	Kaur and Kishore (2012)
*Vigna trinervia*	Tooapee (Thai)	2n = 2x = 22	South and Southeast Asia	Madagascar, South India, Sri Lanka, Myanmar, Malaysia, Sumatra, Java, Timor, and New Guinea	Not available	Tateishi (1985)
*Vigna umbellata*	Rice bean	2n = 2x = 22	Southeast Asia	Fiji, Australia, tropical Africa, Indian Ocean Islands, USA, Honduras, Brazil, and Mexico	Not available	Khadka and Acharya (2009)

The origin of mungbean is considered to be India, where domestication is believed to have first taken place ∼3,500 years ago based on domesticated mungbean diversity data and archeological evidence (Vishnu-Mittre, 1974; Fuller and Harvey, 2006). During the early domestication process, mungbean cultivation migrated to other Asian countries and to Africa. Modern cultivated mungbean has resulted from multiple rounds of domestication and selection, and is currently distributed throughout southern and eastern Asia, Africa, and Austronesia (Vishnu-Mittre, 1974; Lambrides and Godwin, 2007). Its putative progenitor, *V. radiata* var. *sublobata*, is indigenous to the subtropical and tropical regions of northern and eastern Australia (Lawn and Cottrell, 1988). This plant is widely distributed as a weed across many different areas including southern Africa, southern and eastern Asia, and some Pacific islands of Indonesia and Australia. In general, wild species are reservoirs of useful genes, as these genes are present in wild species but not in currently cultivated germplasm due to a genetic bottleneck that has occurred during domestication and modern breeding (Hawkes, 1977; Doyle, 1988; Tanksley and McCouch, 1997; Kumar et al., 2011). The valuable gene pool from wild species has been used by plant breeders for crop improvement (Kumar et al., 2011). For example, mungbean variety TC1966 is completely resistant to two species of bruchid beetles, *Callosobruchus chinensis* (adzuki bean weevil) and *C. maculatus* (cowpea weevil), which cause major damage to mungbean during storage (Talekar, 1988; Visarathanonth and Promsatit, 1989; Somta et al., 2007). This variety has been used to develop a bruchid-resistant mungbean cultivar (Tomooka et al., 1992). Moreover, genetic linkage maps were constructed using a commercial mungbean cultivar and a wild mungbean accession, providing valuable information about important traits such as seed quality, weathering tolerance, and pest/disease resistance (Lambrides et al., 2000). The use of wild species in breeding has tremendous potential benefits, perhaps representing the key to diversifying plant genetic resources while ensuring food security in the face of population growth, which is currently occurring at a rate of up to 160 people per minute (Hoisington et al., 1999). In addition, several research institutes and universities are currently creating germplasm collections to conserve mungbean genetic resources for food and agriculture.

Due to the importance of mungbean, molecular techniques involving random amplified polymorphic DNA (RAPD), restriction fragment length polymorphism (RFLP), single nucleotide polymorphism (SNP), and simple sequence repeat (SSR) markers have long been applied toward improving mungbean, with a focus on yield, nutritional improvement, and disease resistance; these techniques are that could be useful for analyzing germplasm and genetic diversity and for constructing linkage maps. Recently, both cultivated mungbean (*V. radiata*) and a wild relative of mungbean (*V. sublobata*) have been sequenced, along with the transcriptome sequences of 22 *Vigna* accessions of 18 species. These studies have provided insights into the evolution of leguminous species and legume genomics, which may help accelerate the genetic improvement of mungbean (Kang et al., 2014). The objectives of this review are as follows: to provide an overview of the current status of mungbean germplasm collections in different geographical regions; to explore the current progress in structural genomic studies, such as molecular analyses aimed at evaluating genetic diversity in mungbean, linkage map construction for detecting quantitative trait loci (QTLs), and genomic studies of mungbean; to discuss the potential of translational genomics in mungbean and other less-studied *Vigna* species for crop improvement; and to summarize the current status of mungbean projects and future directions to increase the average yields of mungbean.

## Mungbean Genetic Resources

Mungbean genetic resources are maintained at different centers throughout the globe, including the following: the University of the Philippines; AVRDC-The World Vegetable Center, Taiwan; the Institute of Crop Germplasm Resources of the Chinese Academy of Agricultural Sciences; National Bureau of Plant Genetic Resources of the Indian Council of Agricultural Research; and the Plant Genetic Resources Conservation Unit of the University of Georgia, USA (Ebert, 2013). In addition, the University of The Philippines and the Rural Development Administration (RDA), Korea, holds duplicates of parts of the mungbean germplasm collection of AVRDC-The World Vegetable Center.

To enable efficient use of genetic resources and to increase access for breeders, mungbean core collections have been established in countries including China, India, the USA, and Korea. Very recently, AVRDC-The World Vegetable Center developed a core collection comprising 1,481 accessions and a mini-core comprising 296 accessions (Schafleitner et al., 2015). The core collection was developed based on phenotypic characterization, while the mini-core was developed by molecular characterization using 20 SSR markers.

In addition to these mungbean resources, researchers have begun utilizing mungbean-related species in crop improvement programs. For example, *V. mungo* has been used as a source of traits such as yellow mosaic disease resistance for transfer into mungbean. AVRDC-The World Vegetable Center currently holds the world’s largest collection of *Vigna* germplasm, comprising 12,153 accessions (**Table 2**), which represent an important resource for interspecific hybridization.

**Table 2:** Major *Vigna* spp. in the collection at AVRDC-The World Vegetable Center.

*Vigna* species	Number of accessions
*Vigna radiata*	6,742
*Vigna mungo*	853
*Vigna umbellata*	370
*Vigna unguiculata*	1,587
*Vigna angularis*	2,376

## Early Structural Genomics Studies

### First Generation Sequencing Resources

DNA markers have been utilized for germplasm evaluation, genetic diversity and phylogenetic analysis, high-density linkage map construction, genome mapping, and marker-assisted selection to investigate structural genomics in crop plants (Muthamilarasan and Prasad, 2015). Currently, a vast amount of genomic resources is available at NCBI^1^ for mungbean including ESTs (expressed sequence tags) and GSS (genomic survey sequences). However, in earlier mungbean studies, RFLP and RAPD markers were commonly utilized using Sanger sequencing approaches, when the availability of mungbean sequence information was insufficient (Chavan and Gacche, 2014). RFLP probes were used in these studies because these markers are co-dominant and their use yields reproducible results. Young et al. (1992) performed the first study applying RFLP technology to mungbean to analyze the genetics of bruchid resistance. In this study, 153 RFLP markers were mapped to 14 linkage groups covering 1,295 centiMorgans (cM), with an average distance of 9.3 cM between markers. Subsequently, specific genomic regions were examined for seed weight QTLs in cowpea (*V. unguiculata*) and mungbean (Fatokun et al., 1992). The initial linkage map of mungbean was developed from RFLP markers based on clones from mungbean, cowpea (*V. unguiculata*), soybean (*Glycine max*), and common bean (*Phaseolus vulgaris*), and an interspecific hybrid population generated from a cross between *V. radiata* ssp. *radiata* and *V. radiata* ssp. *sublobata.* The map consists of 171 markers covering a total of 1,570 cM on 11 linkage groups, with an average interval of 9 cM between loci, as well as several important traits such as seed size, resistance to powdery mildew, and resistance to seed bruchids (Menancio-Hautea et al., 1992). The genetic relationships between mungbean and cowpea were also investigated by establishing the linkage arrangement of RFLPs. A genetic linkage map constructed using a set of probes derived from cowpea, common bean, mungbean, and soybean *Pst1* genomic libraries revealed that these species share several large linkage blocks and exhibit a high degree of similarity in terms of nucleotide sequence (Menancio-Hautea et al., 1993). Similarly, comparative mapping among three legumes, mungbean, cowpea, and common bean was performed using RFLP maps based on a common set of DNA clones, demonstrating extensive linkage conservation among the three genomes (Boutin et al., 1995). Significant collinearity of synteny blocks among adzuki bean, mungbean, and cowpea was also demonstrated, as the same sets of RFLP markers were localized to the same linkage groups along the respective linkage maps (Kaga, 1996).

Since the early and mid-1990s, RAPD analysis has been successfully performed in several crop plants including wheat (Vierling and Nguyen, 1992; Joshi and Nguyen, 1993), coffee (Orozco-Castillo et al., 1994), *Brassica* (dos Santos et al., 1994), rice (Virk et al., 1995), and banana (Bhat and Jarret, 1995). Since the RFLP approach is laborious and costly, as it requires the use of specific probes for target sequences, as well as radioactive isotopes, an alternative method, the PCR-based RAPD technique, has been used to identify and characterize genotypes (Welsh and McClelland, 1990; Williams et al., 1990). Compared to RFLP, RAPD is simple and fast, involving no radioactive labeling of probes and no specific sequences (Ho et al., 1995). The first RAPD study of mungbean was conducted by Kaga et al. (1996) to determine the genetic variability among 23 accessions of five species of wild and cultivated plants in the subgenus *Ceratotropis*, including *V. angularis, V. umbellata, V. radiata, V. aconitifolia*, and *V. mungo*. Early RAPD analyses in mungbean have focused on examining genotypic diversity and genetic similarity for crop improvement (Bisht et al., 1998; Santalla et al., 1998). By integrating both RFLP and RAPD techniques to detect genetic polymorphism, a linkage map was developed from the F_2_ mapping populations of *V. radiata* ssp. *radiata* and *V. radiata* ssp. *sublobata*, with 52 RFLP and 56 RAPD markers localized to 12 linkage groups (Lambrides et al., 2000). The linked markers cover a total distance of 758.3 cM. As a common set of probes from a published map was used (Menancio-Hautea et al., 1992), the map was partially confirmed by comparing the linkage order and distances of the markers. In addition, a genetic map was constructed using a recombinant inbred population derived from the previously examined F_2_ individuals, consisting of 115 markers in 12 linkage groups covering a distance of 691.7 cM. Furthermore, additional efforts were made to map genes responsible for bruchid resistance based on genetic linkage maps constructed using RFLP and RAPD markers (Kaga and Ishimoto, 1998), to make a better saturated linkage map using RFLP markers alone (Humphry et al., 2002), or for assessment of genetic diversity in mungbean using a combination of RAPD and inter simple sequence repeat (ISSR) profiles (Chattopadhyay et al., 2005). Numerous studies have focused on molecular mapping and locating QTLs for traits in mungbean based on RFLPs (Humphry et al., 2005). However, despite great efforts over the past years, it has been difficult to resolve the 11 linkage groups and to saturate the map without the use of high-throughput SSR and SNP markers (Boopathi, 2013).

### Sequence-Based Genetic Markers

Simple sequence repeat markers are co-dominant, PCR-based markers that are easy to generate, highly polymorphic and effectively used to detect genetic variation based on repeat-lengths. In 1999, the first SSR markers for mungbean were reported by Yu et al. (1999) based on a search of the GenBank database, revealing six SSR sequences with five different types of motifs, including di-, tri-, and tetra-nucleotide repeats (AT)n/(TA)n, (ATT)n/(AAT)n, (GGC)n/(GCC)n, (AGGG)n/(AGGG)n and (CTTT)n/(AAAG)n, in a total length of 67.1 kb. Moreover, using 5′ anchored degenerate primers isolated by a library-enrichment protocol, 23 and 15 microsatellites consisting of di-nucleotide and tetra-nucleotide sequences were revealed, with an observed heterozygosity of 0 to 0.9048 and 0 to 0.561, respectively (Kumar et al., 2002a,b). Gwag et al. (2006) identified seven polymorphic microsatellite loci based on polymorphism screening of 93 designed primer pairs in a panel of 10 mungbean accessions. These markers were characterized which produced 2–5 alleles in 34 mungbean accessions with the observed and expected heterozygosity values from 0 to 0.088 and from 0.275 to 0.683, respectively. Compared to other legume crops, a few SSR markers are available for mungbean. The success of generating a genome map of black gram based on adzuki markers (due to their close phylogenetic relationship) has led to the use of adzuki bean SSR markers in mungbean (Sangiri et al., 2007). Of the 78 adzuki markers examined, 27 were useful for screening polymorphisms in mungbean. Nineteen primers designed based on these markers positioned on each linkage group were used to examine 415 cultivated, 189 wild, and 11 intermediate mungbean accessions. In this study, a higher number of alleles was detected in wild mungbean than in cultivated mungbean, and the SSR marker allelic diversity differed depending on the region (Sangiri et al., 2007). Several studies aimed at finding a novel set of SSR markers in mungbean (Somta et al., 2008; Seehalak et al., 2009). Initial work was performed to develop markers through high-throughput sequencing (Tangphatsornruang et al., 2009). A total of 1,493 microsatellite regions were isolated from 454 pyrosequencing shotgun reads, and among the 192 pairs of SSR primers evaluated in 17 mungbean accessions, polymorphism was detected at 60 loci, ranging from two to six alleles (average of 2.683) per locus. The polymorphism information content (PIC) values ranged from 0.0555 to 0.6907, which is similar to previous findings (Somta et al., 2008; Seehalak et al., 2009). Moreover, the SSRs could potentially be used to construct linkage maps and for genetic improvement of mungbean (Tangphatsornruang et al., 2009); however, all of the SSRs were not associated with linkage groups.

Simple sequence repeat markers have been widely used to identify disease resistance QTLs in legume species, which could be used in marker-assisted selection in crops. Therefore, studies identifying QTL regions and SSR markers conferring resistance to powdery mildew and Cercospora leaf spot in mungbean (based on partial linkage maps with few linkage groups) were conducted by Kasettranan et al. (2010) and Chankaew et al. (2011), respectively. Zhao et al. (2010) developed a high-density genetic linkage map by combining 76 RFLP markers reported by Humphry et al. (2002) with 103 new loci, including 97 SSRs, four RAPDs, and two sequence tag sites (STSs). The resulting linkage map comprises 179 loci spanning 1,831.9 cM, with an average marker interval of 10.2 cM. High homology between mungbean and adzuki bean was revealed by comparing orthologous linkage groups (Zhao et al., 2010). Finally, the 11 initial linkage groups, covering 727.6 cM, were resolved by Isemura et al. (2012) using 237 SSR markers from mungbean and related species such as adzuki bean, mungbean, cowpea, and common bean, as well as 193 EST (expressed sequence tag)-SSR markers from soybean. The resulting map is the first to cover 11 linkage groups, corresponding to the number of haploid mungbean chromosomes, including 105 QTLs and genes for 38 domestication-related traits. The markers identified in this study are useful for the genetic improvement of mungbean due to the availability of sequence information. Furthermore, previously mapped genes and QTLs have been corrected or re-confirmed by Isemura et al. (2012), such as the following: the bruchid resistance gene *Br1*, which was placed in LG2 (Young et al., 1992; Kaga and Ishimoto, 1998) based on work by Menancio-Hautea et al. (1993), was repositioned to LG2; the 100-seed weight QTLs investigated by Fatokun et al. (1992), which were previously mapped to LG vi and LG ii, were repositioned to LG1 (*Sd100wt5.1.2*) and LG8 (*Sd100wt5.8.1*), respectively; and the gene controlling black mottle on the seed coat was mapped to LG2 by Lambrides et al. (2000) but repositioned to LG4. However, the seed weight QTLs revealed by Humphry et al. (2005) could not be used for comparison due to the different types of markers used.

## Advancements in Next-Generation Sequencing (NGS)

With the advancement in NGS, researchers have focused on finding SNPs to be used as genetic markers, as SNPs are co-dominant, single-locus, biallelic markers that are abundant and ubiquitous throughout the genome and are readily used for genotyping (Brumfield et al., 2003). Moe et al. (2011) presented fundamental insights into functional annotation in the mungbean genome, including an overview of its gene content. Two varieties of mungbean, ‘Seonhwanokdu’ (accession *VC1973A*) and ‘Jangannokdu,’ were subjected to 454 sequencing due to their respective susceptibility and resistance to stink bug (*Riptortus clavatus*) and adzuki bean weevil (*Callosobruchus chinensis*). A total of 150,159 and 142,993 reads were produced, generating 5,254 and 6,372 contigs greater than or equal to 500 bp, with an average length of 833 and 853 bp for *VC1973A* and Jangannokdu, respectively. A total of 41.34 and 41.74% unigenes were functionally annotated to known sequences and classified into 17 and 18 FunCat functional categories for *VC1973A* and Jangannokdu, respectively, including metabolism, energy, cell cycle and DNA processing, transcription, protein synthesis, and so on. The authors then performed large-scale discovery of molecular markers: 1,334 (*VC1973A*) and 1,630 (Jangannokdu) microsatellite repeat motifs were counted, and 2,098 highly confident sequence variations were revealed that could be utilized as SNP markers. These findings represent a valuable resource for functional genomics studies aimed at improving mungbean breeding efforts and increasing the marker density of linkage maps.

In recent years, as sequencing has become increasingly popular due to the rapid development of NGS technologies, genome sequences have become available for crop species. Many instruments are used for high-throughput sequencing, including SOLiD/Ion Torrent PGM from Life Science, Genome Analyzer/HiSeq 2000/MiSeq from Illumina, and 454 FLX Titanium/GS Junior by Roche (Liu et al., 2012). Until the early 2000s, when Illumina launched Illumina HiSeq 2000, mungbean SNPs were detected using 454 GS FLX pyrosequencing. As Illumina HiSeq 2000 can be used to produce longer and more accurate contigs compared to Roche 454 (Luo et al., 2012), Van et al. (2013) used this system for *in silico* identification of mungbean markers from two mungbean cultivars, *VC1973A* and Gyeonggijaerae 5 (accession *V2984*); *V2984* is susceptible to mungbean mottle virus, Cercospora leaf spot, and powdery mildew (Hong et al., 1983). The authors prepared a shotgun/paired-end library (500 bp insert) and sequenced more than 40.0 billion bp from both cultivars at a depth of 72x. After *de novo* assembly of contigs and alignment of *VC1973A* and *V2984* reads, 265,001 homozygous SNPs were identified, 65.9% of which were transitions and 34.1% of which were transversions. To confirm the *in silico* SNPs, the common bean ESTs were mapped onto *VC1973A* contigs, revealing approximately 80% sequence similarity between the two species. These findings represent a substantial molecular resource for developing a high-density genetic map and for examining genetic diversity in mungbean.

An important milestone in *Vigna* genomic research was reached in 2014 with the completion of *de novo* sequencing of *VC1973A*, its polyploid relative *V. reflexo*-*pilosa* var. *glabra* (accession *V1160*), and its wild relative *V. radiata* var. *sublobata* (accession *TC1966*), as well as the *de novo* assembly of RNA-seq data from of 22 accessions of 18 *Vigna* species, facilitating advances in genomic research into the subgenus *Ceratotropis* and providing insights into the evolution within *Vigna* species. Kang et al. (2014) sequenced the mungbean genome using Illumina HiSeq 2000 and GS FLX+, generating 2,748 scaffolds with an N50 length of 1.62 Mb on a 431 Mb map (∼80% of the 579 Mb estimated genome size). Sequence analysis revealed 22,427 high-confidence protein-coding genes and 160 *Vigna* gene clusters. Furthermore, a high-density SNP linkage map was constructed using 1,321 genotyping-by-sequencing (GBS) SNP markers covering all 11 linkage groups from an F_6_ population of 190 recombinant inbred lines (RILs) based on a cross between *VC1973A* and the Korean landrace *V2985*. Previously, only low-resolution linkage maps were produced due to the limited number of available markers; however, the high-density genetic map produced in this study has an N50 length of 35.4 Mb, covering a physical length of 314 Mb, which corresponds to 73% of the total mungbean genome. The domestication history of mungbean was traced by comparing sequence variants, such as SNPs and insertions/deletions (INDELs), in wild and cultivated mungbean, especially non-synonymous SNPs in exonic regions related to domestication-related traits. Kang et al. (2014) compared wild and cultivated mungbean genomes, revealing 2,922,833 SNPs at a frequency of 6.78 per kb. A total of 63,294 SNPs were detected in coding sequence (CDS) regions, 30,405 of which were non-synonymous, while 55,689 out of 342,853 INDELS were inserted/deleted bases located within genic boundaries caused by frameshifts in 1,057 genes. A large set of SSR markers (200,808 SSRs) will enhance future studies aimed at identifying the interaction between markers and QTLs, including those involved in biotic and abiotic stress responses.

## Progress in Transcriptomic Marker Development

In recent years, extensive efforts have been made to develop EST-SSR markers in mungbean for functional genomics studies. SSR markers derived from ESTs are powerful not only because they are present in expressed regions of the genome, but also because EST sequences of many crop species are deposited in databases, providing a fast, cost-effective way to develop markers. Isemura et al. (2012) constructed a genetic linkage map using EST-SSR markers from soybean. However, due to the availability of ESTs in two mungbean cultivars (*VC1973A* and Jangannokdu), EST-SSRs have since been identified in the mungbean genome (Gupta et al., 2014). Using 12,596 EST sequences from mungbean genotype ‘Jangannokdu’ generated by Moe et al. (2011), SSRs were retrieved through data mining, including 1,848 EST sequences carrying 2,299 SSR motifs. Of these ESTs, 1,738 (94%) were simple SSRs (perfect and imperfect) and 110 (6%) were compound SSRs. Among the repeat motifs detected, tri-nucleotide motifs (48%) were the most abundant, followed by di-nucleotide motifs (25%), tetra-nucleotide motifs (15%), hexa-nucleotide motifs (7%), and penta-nucleotide motifs (5%). A total of 97 PCR primers were designed based on these EST-SSRs and were successfully amplified in two mungbean cultivars, TM96-2 and TARM-18, revealing that ∼45 and 55% of the SSR motifs were located in CDS and untranslated regions (UTRs), respectively. In addition, 27 randomly selected genic SSR markers were used to analyze the genetic diversity among 20 mungbean accessions. Polymorphism was observed in 21 (78%) genic SSR markers, with a PIC value of 0.34. Chavan and Gacche (2014) also identified EST-SSR markers based on available sequences from the NCBI database for mungbean, including 829 ESTs, 83 GSS (genomic survey sequences), and 2,903 nucleotides. In 2015, to enhance the efficiency of SSR isolation, SSR-enriched libraries were constructed using six genotypes of mungbean (ACC41, VC1973A, V2709, C01478, C01558, and C01579), and a total of 308,509 SSRs (56.9% simple and 43.1% compound) were discovered (Wang et al., 2015). In both studies, the most frequently detected motifs were AC/GT and AAC/GTT.

Due to the importance of functional SSR markers for enhancing gene discovery and elucidating gene function, transcriptome sequencing has been utilized for SSR mining in many plant species, including jatropha (Jain et al., 2014), cabbage (Ding et al., 2015), castor bean (Qiu et al., 2010), and sesame (Zhang et al., 2012). Chen et al. (2015) performed transcriptome sequencing of mungbean by Illumina paired-end sequencing to characterize and validate SSR markers derived from *in silico* EST-SSR markers, complementing a previous study performed by 454 Sequencing (Moe et al., 2011). A total of 10.3 GB of Illumina reads were assembled into 48,693 unigenes, which were searched against the NCBI non-redundant (Nr), Swiss-Prot, Kyoto Encyclopedia of Genes and Genomes (KEGG), and EuKaryotic Ortholog Groups (KOG) databases. Sequences from the Nr sequence database showed significant similarity to 25,820 (53%) known proteins. All of the assembled unigenes were classified into gene ontology categories, Swiss-Prot categories, KOG functional categories, and KEGG pathways for functional validation. Based on these unigenes, potential SSR markers were mined by surveying sequence repeat and a total of 13,134 EST-SSRs were identified; the most abundant motif was mono-nucleotide A/T repeats. To validate the EST-SSR markers, 200 SSR sites were randomly chosen to design primers used to detect polymorphism in eight mungbean accessions. A total of 129 primer pairs based on these markers were successfully used for PCR amplification, including 66 polymorphic and 97 monomorphic markers. Of these primers, 66 primers successfully produced amplicons and were detected to be polymorphic among 31 mungbean accessions. The possible functions of these validated EST-SSRs were identified, most of which were associated with hypothetical protein-coding genes from common bean and soybean. These recent studies substantiate the importance of using EST-SSR markers to create more variability, thereby advancing linkage and QTL mapping for traits of interest, which can be further used for mungbean improvement.

## Translational Genomics

Improvements in sequencing technology have led to the sequencing of the complete genomes of many crop species, which has opened the door for in-depth studies of structural and functional features, with the ultimate aim of crop improvement. Translational genomics tools have made plant breeding easier and more effective by enabling researchers and breeders to exchange genetic and genomic information among species (Gepts et al., 2005; Stacey and VandenBosch, 2005). For example, the development of common legume markers has facilitated translational genomic studies in legume species, especially species lacking sequence information (Gepts et al., 2005). Therefore, translational genomics has also been referred to as genomics-assisted breeding (GAB; Varshney et al., 2005). Genome sequences are currently available for mungbean, but QTL mapping of this crop has not yet been reported. Comparative analysis between mungbean and soybean would assist mungbean QTL mapping, as SoyBase^2^ (the USDA-ARS soybean genetic database) contains over 1,000 QTL sequences for more than 90 agronomically important traits.

Information from previously analyzed species can be utilized for other species, such as model systems to crops, a concept referred to as translational genomics (Varshney et al., 2015). Due to the completion of genome sequencing of several legume species, comparative analysis represents a powerful tool that can be used to support translational genomics studies. Using this technique, genomic knowledge (such as molecular markers) can be applied to crops that are poorly understood, ultimately leading to practical crop breeding and improvement strategies (Varshney et al., 2015). To conduct systematic translational genomic analysis, studies comparing the genome organization of model versus crop species are needed to supplement the available genomic data (Bordat et al., 2011). The potential of translational genomics has been revealed in early studies of legumes, as this technique benefits the analysis of less-studied crops. Based on sequence-based comparisons, Mudge et al. (2005) revealed orthologous and paralogous relationships of the genomic regions harboring soybean nematode resistance genes, *rhg1* and *rhg4*, between soybean and *Medicago truncatula*. Moreover, large-scale comparisons between *M. truncatula* and alfalfa were conducted to elucidate their syntenic relationships, chromosome relationships, and duplication histories (Cannon et al., 2006). Furthermore, several studies have focused on direct crop improvement in legume species based on information from well-studied plants. For example, Kwak et al. (2008) identified candidate genes responsible for determinacy and photoperiod sensitivity traits in common bean (*P. vulgaris*) using homologs in *Arabidopsis thaliana* floral regulatory genes, and Yang et al. (2008) identified a putative resistance gene in alfalfa (*M. sativa*) based on map-based cloning of *RCT1*, a host resistance (*R*) gene in *M. truncatula*, which conferred resistance in alfalfa cultivars. Multiple-legume species macrosynteny comparisons were also performed between cowpea and soybean, and cowpea and *M. truncatula*, based on the genetic map of cowpea. The availability of genome sequences across legume species including *M. truncatula* (Young et al., 2011), *Lotus japonicas* (Sato et al., 2008), soybean (Schmutz et al., 2010), pigeon pea (Varshney et al., 2012), chickpea (Varshney et al., 2013), mungbean (Kang et al., 2014), common bean (Schmutz et al., 2014), and adzuki bean (Kang et al., 2015) have enabled extensive comparative genomics across species, including genomic contents and gene orders. For example, based on a search for reciprocal homologs among all pea gene sequences and genes from three sequenced legume species, *M. truncatula, L. japonicas*, and *G. max*, a total of 5,460 unigenes were positioned on the functional map. A detailed comparison of microsynteny among the species investigated revealed that a candidate gene for the hypernodulation mutation *nod3* in pea is likely to be a homolog of *Pub1*, an *M. truncatula* gene involved in regulating nodulation (Bordat et al., 2011). In addition, soybean flowering genes were detected by searching for homologs of flowering regulatory genes in *Arabidopsis* (Jung et al., 2012), providing a framework for understanding soybean flowering, including flowering pathways and evolutionary mechanisms.

**FIGURE 1:**
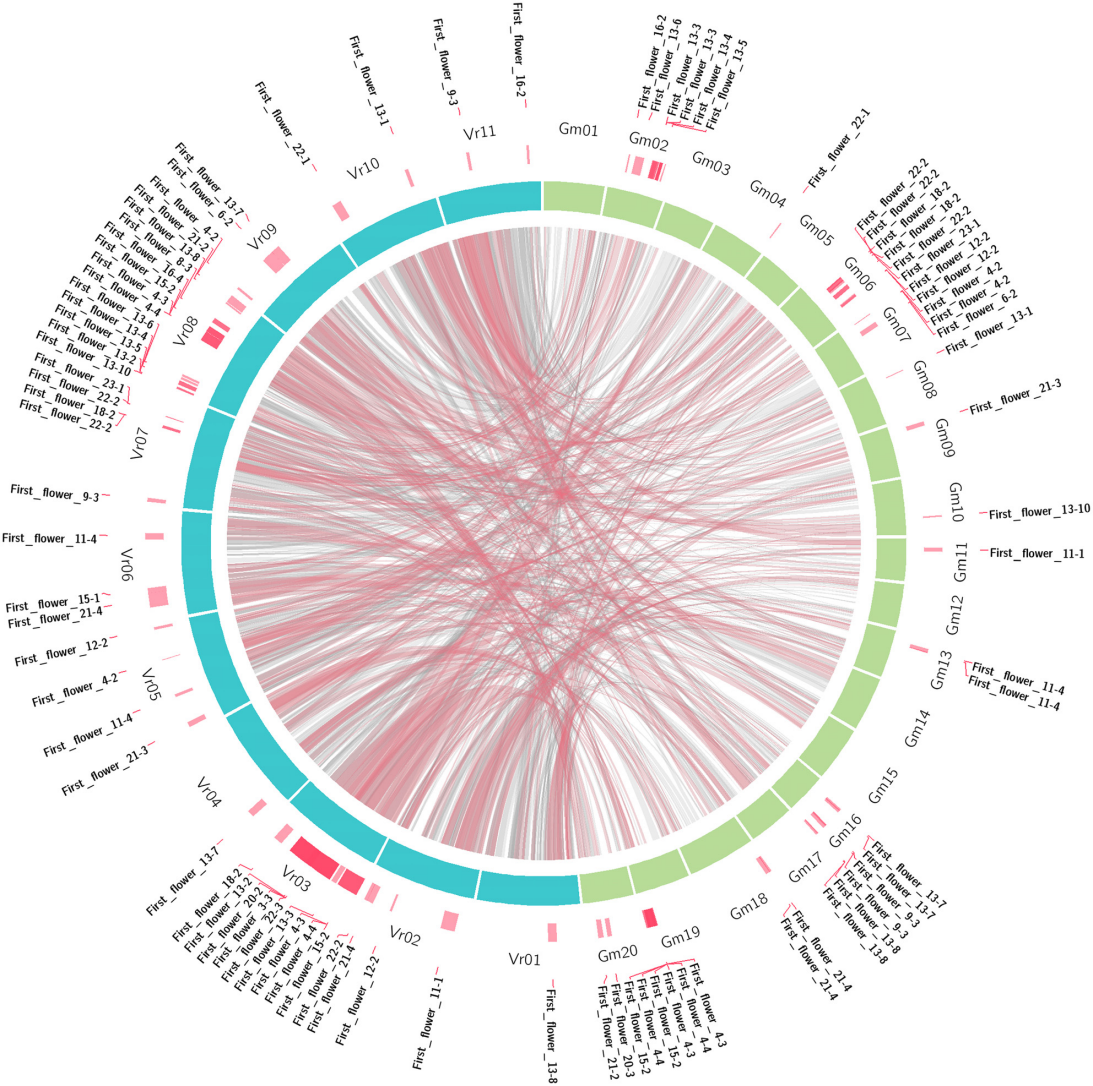
**Comparative analysis of the first flower (days to first flowering) QTLs between mungbean and soybean**.

Recent efforts to identify mungbean flowering genes have been conducted through genome-wide comparative analysis. Of the 207 *Arabidopsis* genes known to be involved in flowering pathways, 129 are homologous to mungbean genes, including five that are also homologous to soybean flowering genes (Kim et al., 2014). Moreover, some of these genes were localized close to SSR markers on a previous genetic linkage map by Isemura et al. (2012). This type of study enables researchers to uncover QTLs associated with specific traits. Based on sequence data from soybean and mungbean and QTLs from soybean^1^ controlling major agronomic traits (Chen et al., 2007), putative mungbean QTLs were also identified (**Table 3**). These major soybean agronomic traits include seed protein content, seed oil content, seed oil-to-protein ratios, pod number, seed weight per plant, seed weight, plant height, pod maturity, leaflet length, leaflet width, and days to first flowering. A circos in **Figure 1** shows comparative analysis of first flower QTLs between mungbean and soybean as one of the examples. Of the 1,650 soybean QTLs underlying these 114 traits, a total of 1,089 QTLs were identified in mungbean (**Table 4**). The number of QTLs detected in mungbean is reasonable, as soybean has undergone two rounds of duplication, whereas mungbean has only undergone one round (Kang et al., 2014). Moreover, extensive conservation of synteny was observed between mungbean and soybean (**Figure 1**). Kang et al. (2014) performed the comparative analysis between mungbean and soybean by aligning the mungbean genome sequences against the soybean genome, revealing that synteny locks containing QTLs responsible for seed size/germination and bruchid resistance matched soybean synteny blocks containing SSR markers associated with seed weight and nematode resistance QTLs. So far, only few studies have focused on aspects of QTLs and potential candidate gene discovery in mungbean regarding major agronomic traits. The current information provides a good starting point for future molecular biological research in mungbean leading to the identification of target genes.

**Table 3:** Number of QTLs identified in mungbean by comparative analysis with soybean QTLs.

Trait	Soybean QTLs	Mungbean QTLs
First flower	104	54
Leaflet length	66	53
Leaflet width	61	55
Plant height	268	171
Pod maturity	196	142
Pod number	59	40
Seed oil	236	178
Seed oil to protein ratios	16	0
Seed protein	356	140
Seed weight	272	245
Seed weight per plant	16	11

**Table 4:** Available online genomic resources for legume crops.

Species	Database	URL
*Lotus japonicus*	Lotus japonicus genome assembly build 2.5	http://www.kazusa.or.jp/lotus/
*Glycine max*	phytozome 10.2	http://phytozome.jgi.doe.gov/soybean
*Medicago truncatula*	PlantGDB	www.plantgdb.org/MtGDB/
*Cajanus cajan*	LIS - Legume Information System	http://legumeinfo.org/gbrowse_cajca1.0
*Phaseolus vulgaris*	phytozome 10.2	http://phytozome.jgi.doe.gov/commonbean
*Cicer arietinum*	Legume Information system	http://cicar.comparative-legumes.org/
*Vigna radiata*	Mungbean Genome Jbrowse	http://plantgenomics.snu.ac.kr/
*Vigna angularis*	Adzuki bean Genome Jbrowse	http://plantgenomics.snu.ac.kr/

## Future Directions

Mungbean was previously considered to be an orphan crop due to the limited number of available genomic resources compared to other legume crops. However, as mungbean has become an important crop in many Asian countries due to its valuable nutritional composition, major studies focusing on increasing yield and quality have been conducted, including studies investigating yellow mosaic disease (Kitsanachandee et al., 2013), bruchid resistance (Mei et al., 2009), Cercospora leaf spot (Chankaew et al., 2011), and other domestication-related traits (Isemura et al., 2012). However, progress in mungbean breeding has been slow due to the lack of genomics research, and relatively little attention has been paid to mungbean since it is widely cultivated in developing countries. However, the release of the complete genome sequence of mungbean is exciting to mungbean researchers and breeders, as it will allow them to accelerate phenotypic screening and, ultimately, breeding for cultivar development through GAB. Furthermore, the advent of next-generation RNA sequencing has enabled researchers to develop a rich pool of genomic resources, including EST-SSR markers (Chen et al., 2015), which may benefit genetic mapping, assessment of genetic diversity, and marker-assisted selection. Since the genome sequence of mungbean has only recently been made available to the public, not much progress has been made on downstream analysis using the mungbean genome sequence. However, putative QTLs identified through *in silico* comparative analysis (**Figure 1**) can be used as a guideline for fine mapping and identifying potential candidate genes related to specific traits. Moreover, a mungbean core collection of 1,481 entries, that have been evaluated for various agronomic traits including primary leaf length, primary leaf width, plant height at flowering, plant height at maturity, days to 50% flowering, pod length, seeds per pod, and 1,000 seed weight (Schafleitner et al., 2015), will be useful for identifying the genomic regions and markers associated with target traits through genome-wide association studies (GWAS), as well as for the detection of candidate genes.

Among Asian *Vigna* species (**Table 1**), only a few have been sequenced, including *V. radiata* (Kang et al., 2014) and *V. angularis* (Kang et al., 2015), while the remaining species have been poorly studied. In order to expand these genomic resources, immense amounts of time, labor, and investment are needed. However, *Vigna* genomic research can be accelerated through translational genomics. For example, the candidate gene approach can be used as a tool for translational genomics. Based on a model species such as *V. radiata*, investigations of genes in other, unsequenced species can be conducted via co-localization of known genes in mungbean, as similar functions or traits can be detected for co-localized genes. For example, the dwarfing gene *Rht* in wheat has the same function as its orthologous gene in *Arabidopsis* (Peng et al., 1999; Salentijn et al., 2007). Similarly, considering the close relationship among legume species, a significant amount of sharing of genes have occurred (Kang et al., 2014), enabling studies of lesser-known *Vigna* crops to be conducted through comparative analysis as a first step in identifying QTLs. Eight legume genome sequences are currently available (**Table 3**), which may facilitate numerous studies aimed at crop improvement of *Vigna* species.

Due to its small genome size, mungbean can be regarded as a model plant among *Vigna* species. Therefore, in-depth studies of the regulatory mechanisms underlying stress or environmental responses are needed to help develop crops that can withstand changing weather patterns and to enable them to grow in harsh environments such as those found in Sub Saharan Africa. This would help in further expansion of mungbean worldwide. Collecting and resequencing wild mungbean from different geographical areas would help researchers investigate allelic variation in beneficial traits that can be mined from wild mungbean. Notably, a wild mungbean genome sequence was released by Kang et al. (2014). Utilizing genomic resources is a promising route for translational genomics studies (Varshney et al., 2015), and this new resource opens the door to genomic research in other *Vigna* species.

## Author Contributions

SK wrote the manuscript, and RN wrote a part of the manuscript and revised it. JL conducted the bioinformatics analyses and designed the figure. SL coordinated the manuscript.

## Conflict of Interest Statement

The authors declare that the research was conducted in the absence of any commercial or financial relationships that could be construed as a potential conflict of interest.

## Footnotes

1 www.ncbi.nlm.nih.gov/

2 www.soybase.org/

## References

[B1] ACC/SCN. (2001). “What works? A review of the efficacy and effectiveness of nutrition interventions,” in *United Nations Administrative Committee on Coordination/Sub-Committee on Nutrition* eds Allen L. H. Gillespie S. R. (Manila: Geneva in collaboration with the Asian Development Bank) 43–53.

[B2] Adsule R. N. (1996). “Moth bean (*Vigna aconitifolia* (Jacq.) Marechal),” in *Food and Feed from Legumes and Oilseeds* eds Nwokolo E. Smartt J. (New York, NY: Springer) 203–205. 10.1007/978-1-4613-0433-3_21

[B3] Agboola A. A. Fayemi A. A. A. (1972). Fixation and excretion of nitrogen by tropical legumes. *Agron. J.* 64 409–412. 10.2134/agronj1972.00021962006400040001x

[B4] Aitawade M. M. Sutar S. P. Rao S. R. Malik S. K. Yadav S. R. Bhat K. V. (2012). Section ceratotropis of subgenus ceratotropis of *Vigna* (Leguminosae–Papilionoideae) in India with a new species from Northern Western Ghats. *Rheedea* 22 20–27.

[B5] Anonymous. (2010). *Mungbean Production Guideline.* Document, Department of Agriculture, Forestry and Fisheries Pretoria, Cape Town Available at: http://www.nda.agric.za/docs/Brochures/MbeanpGUDELINS.pdf

[B6] Arumuganathan K. Earle E. D. (1991). Nuclear DNA content of some important plant species. *Plant Mol. Biol. Rep.* 9 208–218. 10.1007/BF02672069

[B7] AVRDC. (2015). *Door Opens to Myanmar. AVRDC East and Southeast Latest News* Shanhua Available at: http://avrdc.org/door-opens-to-myanmar/

[B8] Bhat K. V. Jarret R. L. (1995). Random amplified polymorphic DNA and genetic diversity in Indian Musa germplasm. *Genet. Res. Crop Evol.* 42 107–118. 10.1007/BF02539514

[B9] Bisht I. S. Mahajan R. K. Kawalkar T. G. (1998). Diversity in greengram (*Vigna radiata* (L.) Wilczek) germplasm collection and its potential use in crop improvement. *Ann. Appl. Biol.* 132 301–312. 10.1111/j.1744-7348.1998.tb05205.x

[B10] Boopathi N. M. (2013). “Mungbean,” in *Genetic Mapping and Marker Assisted Selection: Basics, Practice and Benefits* ed. Paterson A. H. (Chennai, Tamil Nadu: Springer-Verlag) 267–270. 10.1007/978-81-322-0958-4

[B11] Bordat A. Savois V. Nicolas M. Salse J. Chauveau A. Bourgeois M. (2011). Translational genomics in legumes allowed placing in silico 5460 unigenes on the pea functional map and identified candidate genes in *Pisum sativum* L. *G3* 1 93–103. 10.1534/g3.111.000349 22384322 PMC3276132

[B12] Boutin S. R. Young N. D. Olson T. C. Yu Z. H. Shoemaker R. C. Vallejos C. E. (1995). Genome conservation among three legume genera detected with DNA markers. *Genome* 38 928–937. 10.1139/g95-122 18470218

[B13] Brumfield R. T. Beerli P. Nickerson D. A. Edwards S. V. (2003). The utility of single nucleotide polymorphisms in inferences of population history. *Trends Ecol. Evol.* 18 249–256. 10.1016/S0169-5347(03)00018-1

[B14] Bushby H. Lawn R. (1992). Accumulation and partitioning of nitrogen and dry matter by contrasting genotypes of mungbean (*Vigna radiata* L. Wilczek). *Aust. J. Agric. Res.* 43 1609–1628. 10.1071/AR9921609

[B15] Cannon S. B. Sterck L. Rombauts S. Sato S. Cheung F. Gouzy J. (2006). Legume genome evolution viewed through the *Medicago truncatula* and *Lotus japonicus* genomes. *Proc. Natl. Acad. Sci. U.S.A.* 103 14959–14964. 10.1073/pnas.0603228103 17003129 PMC1578499

[B16] Chankaew S. Somta P. Sorajjapinun W. Srinives P. (2011). Quantitative trait loci mapping of Cercospora leaf spot resistance in mungbean, *Vigna radiata* (L.) Wilczek. *Mol. Breed.* 28 255–264. 10.1007/s11032-010-9478-1

[B17] Chattopadhyay K. Ali M. N. Sarkar H. K. Mandal N. Bhattacharyya S. (2005). Diversity analysis by RAPD and ISSR markers among the selected mungbean [*Vigna radiata* (L.) Wilczek] genotypes. *Indian J. Genet. Plant Breed.* 65 173–175.

[B18] Chavan S. P. Gacche R. (2014). Identification and characterization of EST-SSRs in mungbean identification and characterization of EST-SSRs in mungbean. *Webmedcentral* 5:WMC004598 10.9754/journal.wmc.2014.004598

[B19] Chen H. Wang L. Wang S. Liu C. Blair M. W. Cheng X. (2015). Transcriptome sequencing of mung bean (*Vigna radiata* L.) genes and the identification of EST-SSR markers. *PLoS ONE* 10:e0120273 10.1371/journal.pone.0120273 PMC4382333 25830701

[B20] Chen Q. Zhang Z. Liu C. Xin D. Qiu H. Shan D. (2007). QTL analysis of major agronomic traits in soybean. *Agric. Sci. China* 6 399–405. 10.1016/S1671-2927(07)60062-5

[B21] Chotechuen S. (1996). “Breeding of mungbean for resistance to various environmental stresses,” in *Mungbean Germplasm: Collection, Evaluation and Utilization for Breeding Program* eds Srinives P. Kitbamroong C. Miyazaki S. (Bangkok: Proceedings of the Workshop on Mungbean Germplasm) 52–59.

[B22] Ding Q. Li J. Wang F. Zhang Y. Li H. Zhang J. (2015). Characterization and development of EST-SSRs by deep transcriptome sequencing in Chinese cabbage (*Brassica rapa* L. ssp. pekinensis). *Int. J. Genomics* 2015:473028. 10.1155/2015/473028 PMC4609433 26504770

[B23] dos Santos J. B. Nienhuis J. Skroch P. Tivang J. Slocum M. K. (1994). Comparison of RAPD and RFLP genetic markers in determining genetic similarity among *Brassica oleracea* L. genotypes. *Theor. Appl. Genet.* 87 909–915. 10.1007/BF00225784 24190524

[B24] Doyle J. J. (1988). 5S ribosomal gene variation in the soybean and its progenitor. *Theor. Appl. Genet.* 75 621–624. 10.1007/BF00289130

[B25] Ebert A. W. (2013). “Ex situ conservation of plant genetic resources of major vegetables,” in *Conservation of Tropical Plant Species* eds Normah M. N. Chin H. F. Reed B. M. (New York, NY: Springer Press) 373–417.

[B26] Fatokun C. A. Menancio-Hautea D. I. Danesh D. Young N. D. (1992). Evidence for orthologous seed-weight genes in cowpea and mung bean based on RFLP mapping. *Genetics* 132 841–846. 1361476 10.1093/genetics/132.3.841 PMC1205219

[B27] Fuller D. Q. Harvey E. L. (2006). The archaeobotany of Indian pulses: identification, processing and evidence for cultivation. *Environ. Archaeol.* 11 241–268. 10.1179/174963106x123232

[B28] Garg D. D. Arya R. S. Sharma T. Dhuria R. K. (2004). Effect of replacement of sewan straw (*Lasirus sindicus*) by moong (*Phaseolus aureus*) chara on rumen and haemato-biochemical parameters in sheep. *Vet. Pract.* 5 70–73.

[B29] Gepts P. Beavis W. D. Brummer E. C. Shoemaker R. C. Stalker H. T. Weeden N. F. (2005). Legumes as a model plant family. Genomics for food and feed report of the cross-legume advances through genomics conference. *Plant Physiol.* 137 1228–1235. 10.1104/pp.105.060871 15824285 PMC1088316

[B30] Gupta S. K. Bansal R. Gopalakrishna T. (2014). Development and characterization of genic SSR markers for mungbean (*Vigna radiata* (L.) Wilczek). *Euphytica* 195 245–258. 10.1007/s10681-013-0993-0

[B31] Gupta S. Gupta D. S. Tuba Anjum K. Pratap A. Kumar J. (2013). Transferability of simple sequence repeat markers in blackgram (*Vigna mungo* L. Hepper). *Aust. J. Crop Sci.* 7 345–353.

[B32] Gwag J.-G. Chung J.-W. Chung H.-K. Lee J.-H. Ma K.-H. Dixit A. (2006). Characterization of new microsatellite markers in mung bean, *Vigna radiata* (L.). *Mol. Ecol. Notes* 6 1132–1134. 10.1111/j.1471-8286.2006.01461.x

[B33] Hawkes J. G. (1977). The importance of wild germplasm in plant breeding. *Euphytica* 26 615–621. 10.1007/BF00021686

[B34] Ho C. L. Phang S. M. Pang T. (1995). Molecular characterization of *Sargassum polycystum* and S. *siliqosum* (Phaeophyta) by polymerase chain reaction (PCR) using random amplified polymorphic DNA (RAPD) primers. *J. Appl. Phycol.* 7 33–41. 10.1007/BF00003547

[B35] Hoisington D. Khairallah M. Reeves T. Ribaut J. M. Skovmand B. Tabaand S. (1999). Plant genetic resources: what can they contribute toward increased crop productivity? *Proc. Natl. Acad. Sci. U.S.A.* 96 5937–5943. 10.1073/pnas.96.11.5937 10339521 PMC34209

[B36] Hong E. H. Lee Y. H. Kim S. D. Hwang Y. H. Moon Y. H. Ham Y. S. (1983). New disease resistant and high yielding mungbean variety “Seonhwanogdu.” *Res. Rept.* 25 178–180.

[B37] Humphry M. E. Konduri V. Lambrides C. J. Magner T. McIntyre C. L. Aitken E. A. B. (2002). Development of a mungbean (*Vigna radiata*) RFLP linkage map and its comparison with lablab (Lablab purpureus) reveals a high level of collinearity between the two genomes. *Theor. Appl. Genet.* 105 160–166. 10.1007/s00122-002-0909-1 12582573

[B38] Humphry M. E. Lambrides C. J. Chapman S. C. Aitken E. A. B. Imrie B. C. (2005). Relationships between hard-seededness and seed weight in mungbean (*Vigna radiata*) assessed by QTL analysis. *Plant Breed.* 124 292–298. 10.1111/j.1439-0523.2005.01084.x

[B39] Imrie B. C. (1996). “Mung bean,” in *The New Rural Industries: A Handbook for Farmers and Investors* ed. Hyde K. (Canberra: Rural Industries Research and Development Corporation) 355–360.

[B40] Isemura T. Kaga A. Tabata S. Somta P. Srinives P. (2012). Construction of a genetic linkage map and genetic analysis of domestication related traits in mungbean (*Vigna radiata*). *PLoS ONE* 7:e41304 10.1371/journal.pone.0041304 PMC3410902 22876284

[B41] Jain N. Patil G. B. Bhargava P. Nadgauda R. S. (2014). In silico mining of EST-SSRs in *Jatropha curcas* L. towards assessing genetic polymorphism and marker development for selection of high oil yielding clones. *Am. J. Plant Sci.* 5 1521–1541. 10.4236/ajps.2014.511167

[B42] Joshi C. P. Nguyen H. T. (1993). Application of the random amplified polymorphic DNA technique for the detection of polymorphism among wild and cultivated tetraploid wheats. *Genome* 36 602–609. 10.1139/g93-081 8349131

[B43] Jung C.-H. Wong C. E. Singh M. B. Bhalla P. L. (2012). Comparative genomic analysis of soybean flowering genes. *PLoS ONE* 7:e38250 10.1371/journal.pone.0038250 PMC3367986 22679494

[B44] Kaga A. (1996). *Construction and Application of Linkage Maps for Azuki Bean (Vigna angularis).* Ph.D. thesis, Kobe University Kobe.

[B45] Kaga A. Isemura T. Tomooka N. Vaughan D. A. (2008). The genetics of domestication of the azuki bean (*Vigna angularis*). *Genetics* 178 1013–1036. 10.1534/genetics.107.078451 18245368 PMC2248364

[B46] Kaga A. Ishimoto M. (1998). Genetic localization of a bruchid resistance gene and its relationship to insecticidal cyclopeptide alkaloids, the vignatic acids, in mungbean (*Vigna radiata* L. Wilczek). *Mol. Gen. Genet.* 258 378–384. 10.1007/s004380050744 9648742

[B47] Kaga A. Tamooka N. Egawa Y. Hosaka K. Kamijima O. (1996). Species relationships in subgenus Ceratotropis (genus *Vigna*) as revealed by RAPD analysis. *Euphytica* 88 17–24. 10.1007/BF00029261

[B48] Kang Y. J. Kim S. K. Kim M. Y. Lestari P. Kim K. H. Ha B. K. (2014). Genome sequence of mungbean and insights into evolution within *Vigna* species. *Nat. Commun.* 5 5443 10.1038/ncomms6443 PMC4241982 25384727

[B49] Kang Y. J. Satyawan D. Shim S. Lee T. Lee J. Hwang W. J. (2015). Draft genome sequence of adzuki bean, *Vigna angularis*. *Sci. Rep.* 5 8069 10.1038/srep08069 PMC5389050 25626881

[B50] Kasettranan W. Somta P. Srinives P. (2010). Mapping of quantitative trait loci controlling powdery mildew resistance in mungbean (*Vigna radiata* (L.) Wilczek). *J. Crop Sci. Biotechnol.* 13 155–161. 10.1007/s12892-010-0052-z

[B51] Kaur N. Kishore L. (2012). Antioxidant activity of methanolic extract of *Phaseolus trilobus* root powder. *Int. J. Pharm. Pharm. Sci.* 4 1–5.

[B52] Khadka K. Acharya B. D. (2009). *Cultivation Practices of Ricebean.* Nepal: Pokhara.

[B53] Kim S. K. Lee T. Kang Y. J. Hwang W. J. Kim K. H. Moon J.-K. (2014). Genome-wide comparative analysis of flowering genes between *Arabidopsis* and mungbean. *Genes Genomics* 36 799–808. 10.1007/s13258-014-0215-8

[B54] Kitsanachandee R. Somta P. Chatchawankanphanich O. Akhtar K. P. Shah T. M. Nair R. M. (2013). Detection of quantitative trait loci for mungbean yellow mosaic India virus (MYMIV) resistance in mungbean (*Vigna radiata* (L.) Wilczek) in India and Pakistan. *Breed. Sci.* 63 367–73. 10.1270/jsbbs.63.367 24399908 PMC3859347

[B55] Kumar J. Choudhary A. K. Solanki R. K. Pratap A. (2011). Towards marker-assisted selection in pulses: a review. *Plant Breed.* 130 297–313. 10.1111/j.1439-0523.2011.01851.x

[B56] Kumar S. V. Tan S. G. Quah S. C. Yusoff K. (2002a). Isolation of microsatellite markers in mungbean, *Vigna radiata*. *Mol. Ecol. Notes.* 2 96–98. 10.1046/j.1471-8286.2002.00158.x

[B57] Kumar S. V. Tan S. G. Quah S. C. Yusoff K. (2002b). Isolation and characterization of seven tetranucleotide microsatellite loci in mungbean, *Vigna radiata*. *Mol. Ecol. Notes* 2 293–295. 10.1046/j.1471-8286.2002.00239.x

[B58] Kwak M. Velasco D. Gepts P. (2008). Mapping homologous sequences for determinacy and photoperiod sensitivity in common bean (*Phaseolus vulgaris*). *J. Hered.* 99 283–291. 10.1093/jhered/esn005 18316323

[B59] Lambrides C. J. Godwin I. (2007). “Mungbean,” in *Genome Mapping and Molecular Breeding in Plants Pulses, Sugar and Tuber Crops* Vol. 3 ed. Kole C. (Berlin: Springer) 69–90.

[B60] Lambrides C. J. Lawn R. J. Godwin I. D. Manners J. Imrie B. C. (2000). Two genetic linkage maps of mungbean using RFLP and RAPD markers. *Aust. J. Agric. Res.* 51 415–425. 10.1071/AR99052

[B61] Lawn R. J. Cottrell A. (1988). Wild mungbean and its relatives in Australia. *Biologist* 35 267–273.

[B62] Liu L. Li Y. Li S. Hu N. He Y. Pong R. (2012). Comparison of next-generation sequencing systems. *J. Biomed. Biotechnol.* 2012:251364 10.1155/2012/251364 PMC3398667 22829749

[B63] Luo C. Tsementzi D. Kyrpides N. Read T. Konstantinidis K. T. (2012). Direct comparisons of Illumina vs. Roche 454 sequencing technologies on the same microbial community DNA sample. *PLoS ONE* 7:e30087 10.1371/journal.pone.0030087 PMC3277595 22347999

[B64] Mei L. Cheng X. Z. Wang S. H. Wang L. X. Liu C. Y. Sun L. (2009). Relationship between bruchid resistance and seed mass in mungbean based on QTL analysis. *Genome* 52 589–596. 10.1139/G09-031 19767890

[B65] Menancio-Hautea D. Fatokun C. A. Kumar L. Danesh D. Young N. D. (1993). Comparative genome analysis of mungbean (*Vigna radiata* L. Wilczek) and cowpea (*Vigna unguiculata* L. Walpers) using RFLP mapping data. *Theor. Appl. Genet.* 86 797–810. 10.1007/BF00212605 24193874

[B66] Menancio-Hautea D. Kumar L. Danesh D. Young N. D. (1992). “A genome map for mungbean [*Vigna radiata* (L.) Wilczek] based on DNA genetic markers (2N ¼ 2X ¼ 22),” in *Genome Maps* ed. O’Brien S. J. (Cold Spring Harbor, NY: Cold Spring Harbor Laboratory Press) 259–261.

[B67] Moe K. T. Chung J.-W. Cho Y.-I. Moon J.-K. Ku J.-H. Jung J.-K. (2011). Sequence information on simple sequence repeats and single nucleotide polymorphisms through transcriptome analysis of mungbean. *J. Integr. Plant Biol.* 53 63–73. 10.1111/j.1744-7909.2010.01012.x 21205180

[B68] Mudge J. Cannon S. B. Kalo P. Oldroyd G. E. Roe B. A. Town C. D. (2005). Highly syntenic regions in the genomes of soybean *Medicago truncatula* and *Arabidopsis thaliana*. *BMC Plant Biol.* 5:15 10.1186/1471-2229-5-15 PMC1201151 16102170

[B69] Muthamilarasan M. Prasad M. (2015). Advances in *Setaria genomics* for genetic improvement of cereals and bioenergy grasses. *Theor. Appl. Genet.* 128 1–14. 10.1007/s00122-014-2399-325239219 25239219

[B70] Nair R. M. Schafleitner R. Kenyon L. Srinivasan R. Easdown W. Ebert A. (2012). Genetic improvement of mungbean. *SABRAO J. Breed. Genet.* 44 177–190.

[B71] Orozco-Castillo C. Chalmers K. J. Wauh R. Powell W. (1994). Detection of genetic diversity and selective gene introgression in coffee using RAPD markers. *Theor. Appl. Genet.* 8 934–940. 10.1007/bf00225787 24190527

[B72] Parida A. Raina S. Narayan R. (1990). Quantitative DNA variation between and within chromosome complements of *Vigna* species (Fabaceae). *Genetica* 82 125–133. 10.1007/BF00124642

[B73] Peng J. Richards D. E. Hartley N. M. Murphy G. P. Devos K. M. Flintham J. E. (1999). ‘Green revolution’ genes encode mutant gibberellin response modulators. *Nature* 400 256–261. 10.1038/22307 10421366

[B74] Qiu L. Yang C. Tian B. Yang J.-B. Liu A. (2010). Exploiting EST databases for the development and characterization of EST-SSR markers in castor bean (*Ricinus communis* L.). *BMC Plant Biol.* 10:278 10.1186/1471-2229-10-278 PMC3017068 21162723

[B75] Salentijn E. M. J. Pereira A. Angenent G. C. Van Der Linden C. G. Krens F. Smulders M. J. M. (2007). Plant translational genomics: from model species to crops. *Mol. Breed.* 20 1–13. 10.1007/s11032-006-9069-3

[B76] Sangiri C. Kaga A. Tomooka N. Vaughan D. Srinives P. (2007). Genetic diversity of the mungbean (*Vigna radiata*, Leguminosae) genepool on the basis of microsatellite analysis. *Aust. J. Bot.* 55 837–847. 10.1071/BT07105

[B77] Santalla M. Power J. B. Davey M. R. (1998). Genetic diversity in mung bean germplasm revealed by RAPD markers. *Plant Breed.* 117 473–478. 10.1111/j.1439-0523.1998.tb01976.x

[B78] Sato S. Nakamura Y. Kaneko T. Asamizu E. Kato T. Nakao M. (2008). Genome structure of the legume, *Lotus japonicus*. *DNA Res.* 15 227–239. 10.1093/dnares/dsn008 18511435 PMC2575887

[B79] Schafleitner R. Nair R. M. Rathore A. Wang Y. Lin C. Chu S. (2015). The AVRDC–The World Vegetable Center mungbean (*Vigna radiata*) core and mini core collections. *BMC Genomics* 16:344 10.1186/s12864-015-1556-7 PMC4422537 25925106

[B80] Schmutz J. Cannon S. B. Schlueter J. Ma J. Mitros T. Nelson W. (2010). Genome sequence of the palaeopolyploid soybean. *Nature* 463 178–183. 10.1038/nature08957 20075913

[B81] Schmutz J. McClean P. E. Mamidi S. Wu G. A. Cannon S. B. Grimwood J. (2014). A reference genome for common bean and genome-wide analysis of dual domestications. *Nat. Genet.* 46 707–713. 10.1038/ng.3008 24908249 PMC7048698

[B82] Seehalak W. Somta P. Musch W. Srinives P. (2009). Microsatellite markers for mungbean developed from sequence database. *Mol. Ecol. Resour.* 9 862–864. 10.1111/j.1755-0998.2009.02655.x 21564770

[B83] Shanmugasundaram S. Keatinge J. D. H. Hughes J. D. (2009). “Counting on beans: mungbean improvement in Asia,” in *Millions Fed: Proven Successes in Agricultural Development* eds Spielman D. J. Pandya-Lorch R. (Washington, DC: International Food Policy Research Institute) 103–108.

[B84] Somta P. Ammaranan C. Peter A. C. O. Srinives P. (2007). Inheritance of seed resistance to bruchids in cultivated mungbean (*Vigna radiata* L. Wilczek). *Euphytica* 155 47–55. 10.1007/s10681-006-9299-9

[B85] Somta P. Musch W. Kongsamai B. Chanprame S. Nakasathien S. Toojinda T. (2008). New microsatellite markers isolated from mungbean (*Vigna radiata* (L.) Wilczek). *Mol. Ecol. Res.* 8 1155–1157. 10.1111/j.1755-0998.2008.02219.x 21586000

[B86] Stacey G. VandenBosch K. (2005). ‘Translational’ legume biology. Models to crops. *Plant Physiol.* 137 1173. 10.1104/pp.104.900141 PMC1088309 15824278

[B87] Talekar N. S. (1988). “Biology, damage and control of bruchid pests of mungbean,” in *Mungbean: Proceedings of the Second International Symposium* eds Shanmugasundaram S. McLean B. T. (Tainan: AVRDC) 329–342.

[B88] Tangphatsornruang S. Somta P. Uthaipaisanwong P. Chanprasert J. Sangsrakru D. Seehalak W. (2009). Characterization of microsatellites and gene contents from genome shotgun sequences of mungbean (*Vigna radiata* (L.) Wilczek). *BMC Plant Biol.* 9:137 10.1186/1471-2229-9-137 PMC2788553 19930676

[B89] Tanksley S. D. McCouch S. R. (1997). Seed banks and molecular maps, unlocking genetic potential from the wild. *Science* 277 1063–1066. 10.1126/science.277.5329.1063 9262467

[B90] Tateishi Y. (1985). *A revision of the Azuki Bean Group, the Subgenus Ceratotropis of the Genus Vigna (Leguminosae).* Ph.D. thesis, Tohoku University Sendai.

[B91] Tomooka N. Kaga A. Isemura T. Vaughan D. (2010). “*Vigna*,” in *Wild Crop Relatives: Genomic and Breeding Resources* ed. Kole C. (Berlin: Springer-Verlag) 291–311.

[B92] Tomooka N. Lairungruang C. Nakeeraks P. Egawa Y. Thavarasook C. (1992). Development of bruchid-resistant mungbean using wild mungbean germplasm in Thailand. *Plant Breed.* 109 60–66. 10.1111/j.1439-0523.1992.tb00151.x

[B93] Tomooka N. Maxted N. Thavarasook C. Jayasuriya A. H. M. (2002). Two new species, sectional designations and new combinations in *Vigna* subgenus Ceratotropis (Piper) Vedc., (Leguminosae, Phaseoleae). *Kew Bull.* 57 613–624. 10.2307/4110989

[B94] Van K. Kang Y. J. Han K. S. Lee Y. H. Gwang J. G. Moon J. K. (2013). Genome-wide SNP discovery in mungbean by Illumina HiSeq. *Theor. Appl. Genet.* 126 2017–2027. 10.1007/s00122-013-2114-9 23674132

[B95] Varshney R. K. Chen W. Li Y. Bharti A. K. Saxena R. K. Schlueter J. A. (2012). Draft genome sequence of pigeonpea (*Cajanus cajan*), an orphan legume crop of resource-poor farmers. *Nat. Biotechnol.* 30 83–89. 10.1038/nbt.2022 22057054

[B96] Varshney R. K. Graner A. Sorrells M. E. (2005). Genomics-assisted breeding for crop improvement. *Trends Plant Sci.* 10 621–630. 10.1016/j.tplants.2005.10.004 16290213

[B97] Varshney R. K. Kudapa H. Pazhamala L. Chitikineni A. Thudi M. Bohra A. (2015). Translational genomics in agriculture: some examples in grain legumes. *CRC Crit. Rev. Plant Sci.* 34 169–194. 10.1080/07352689.2014.897909

[B98] Varshney R. K. Song C. Saxena R. K. Azam S. Yu S. Sharpe A. G. (2013). Draft genome sequence of chickpea (*Cicer arietinum*) provides a resource for trait improvement. *Nat. Biotechnol.* 31 240–246. 10.1038/nbt.2491 23354103

[B99] Vayupharp B. Laksanalamai V. (2013). Nutrients and anti-nutrients of high chlorophyll – mungbean sprouts as affected by different periods of germination and sprouting stages. *Int. J. Agric. Biol. Eng.* 6 121.

[B100] Vierling R. A. Nguyen H. (1992). Use of RAPD markers to determine the genetic diversity of diploid wheat genotypes. *Theor. Appl. Genet.* 84 835–838. 10.1007/bf00227393 24201483

[B101] Vijayalakshmi P. S. Amirthaveni S. Devada R. P. Weinberger K. Tsou S. C. S. Shanmugasundaram S. (2003). “Enhanced bioavailability of iron from mungbean and its effects on health of school children,” in *Technical Bulletin No. 30 AVRDC Publication 03-559* ed. Kalb T. (Shanhua: AVRDC-The World Vegetable Center) 5–26.

[B102] Virk P. S. Newbury H. J. Jackson M. T. Ford-Lloyd B. V. (1995). The identification of duplicate accessions within a rice germplasm collection using RAPD analysis. *Theor. Appl. Genet.* 90 1049–1055. 10.1007/BF00222920 24173061

[B103] Visarathanonth P. Promsatit B. (1989). “Bruchid loss and control in Thailand,” in *Loss From and Control of Bruchids in Developing Countries, in Proceedings of the 2nd International Symposium On Bruchids and Legumes, Country Report Session* ed. Yoshida T. (Tokyo: Japanese Society of Applied Entomology and Zoology) 44–53.

[B104] Vishnu-Mittre B. (1974). “Palaeobotanical evidence in India,” in *Evolutionary Studies in World Crops: Diversity and Change in the Indian Sub-continent* ed. Hutchinson J. (Cambridge: Cambridge University Press) 3–30.

[B105] Wang L. X. Elbaidouri M. Abernathy B. Chen H. L. Wang S. H. Lee S. H. (2015). Distribution and analysis of SSR in mung bean (*Vigna radiata* L.) genome based on an SSR-enriched library. *Mol. Breed.* 35 25 10.1007/s11032-015-0259-8

[B106] Welsh J. McClelland M. (1990). Fingerprinting genomes using PCR with arbitrary primers. *Nucleic Acids Res.* 18 7213–7218. 10.1093/nar/18.24.7213 2259619 PMC332855

[B107] Williams J. G. K. Kubelik A. R. Livak K. J. Rafalski J. A. Tingey S. V. (1990). DNA polymorphisms amplified by arbitrary primers are useful as genetic markers. *Nucleic Acids Res.* 18 6531–6535. 10.1093/nar/18.22.6531 1979162 PMC332606

[B108] Yang R. S. Tsou S. C. S. (1998). “Mungbean as a potential iron sources in South Asian diets,” in *Proceedings of International Consultation Workshop on Mungbean* ed. Shanmugasundaram S. (Shanhua: AVRDC-The World Vegetable Center) 152–158.

[B109] Yang S. Gao M. Xu C. Gao J. Deshpande S. Lin S. (2008). Alfalfa benefits from *Medicago truncatula*: the RCT1 gene from *M. truncatula* confers broad-spectrum resistance to anthracnose in alfalfa. *Proc. Natl. Acad. Sci. U.S.A.* 105 12164–12169. 10.1073/pnas.0802518105 18719113 PMC2527883

[B110] Yaqub M. Mahmood T. Akhtar M. Iqbal M. M. Ali S. (2010). Induction of mungbean [*Vigna radiata* (L.) Wilczek] as a grain legume in the annual rice-wheat double cropping system. *Pakistan J. Bot.* 42 3125–3135.

[B111] Young N. D. Debellé F. Oldroyd G. E. D. Geurts R. Cannon S. B. Udvardi M. K. (2011). The Medicago genome provides insight into the evolution of rhizobial symbioses. *Nature* 480 520–524. 10.1038/nature10625 22089132 PMC3272368

[B112] Young N. D. Kumar L. Menancio-Hautea D. Danesh D. Talekar N. S. Shanmugasundaram S. (1992). RFLP mapping of major bruchid resistance gene in mungbean (*Vigna radiata*). *Theor. Appl. Genet.* 84 839–844. 10.1007/bf00227394 24201484

[B113] Yu K. Park S. J. Poysa V. (1999). Abundance and variation of microsatellite DNA sequences in beans (*Phaseolus* and *Vigna*). *Genome* 42 27–34. 10.1139/gen-42-1-27

[B114] Zhang H. Wei L. Miao H. Zhang T. Wang C. (2012). Development and validation of genic-SSR markers in sesame by RNA-seq. *BMC Genomics* 13:316 10.1186/1471-2164-13-316 PMC3428654 22800194

[B115] Zhao D. Cheng X. Z. Wang L. X. Wang S. H. Ma Y. L. (2010). Construction of mungbean genetic linkage map. *Acta Agronom. Sinica* 36 932–939. 10.1016/S1875-2780(09)60054-7

